# (*Z*)-3-[(4-Eth­oxy­phen­yl)(hy­droxy)methyl­idene]-1-isopropyl­pyrrolidine-2,4-dione

**DOI:** 10.1107/S1600536810046465

**Published:** 2010-11-17

**Authors:** Shang-Yuan Liu, Hai-Zhen Xu, You-Quan Zhu

**Affiliations:** a College of Chemistry, Tianjin Normal University, 393 Binshuixi Road, Xiqing District, Tianjin 300387, People’s Republic of China; b State Key Laboratory of Elemento-Organic Chemistry, Nankai University, Tianjin 300071, People’s Republic of China

## Abstract

In the title compound, C_16_H_19_NO_4_, a potent new herbicide, the dihedral angle between the benzene and pyrrolidine rings is 11.09 (8)°. Intra­molecular O—H⋯O and C—H⋯O hydrogen bonds are observed.

## Related literature

For the anti­biotic activity of 3-acyl­pyrrolidine-2,4-dione compounds, see: van der Baan *et al.* (1978 ▶); Holzapfel *et al.* (1970 ▶); Mackellar *et al.* (1971 ▶); Rinehart *et al.* (1963 ▶); Sticking (1959 ▶); Wu *et al.* (2002 ▶). For a related structure, see: Ellis & Spek (2001 ▶). For the synthesis, see: Matsuo *et al.* (1980 ▶). For bond-length data, see: Allen *et al.* (1987 ▶).
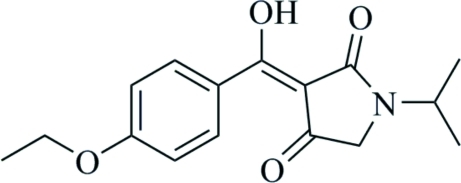


## Experimental

### 

#### Crystal data

C_16_H_19_NO_4_
*M* *_r_* = 289.32
Monoclinic, 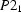

*a* = 11.3390 (15) Å
*b* = 5.3830 (8) Å
*c* = 12.0490 (18) Å
β = 91.581 (7)°
*V* = 735.16 (18) Å^3^
*Z* = 2
Mo *K*α radiation
μ = 0.09 mm^−1^
*T* = 113 K
0.42 × 0.26 × 0.10 mm

#### Data collection

Rigaku Saturn724 CCD diffractometer
Absorption correction: multi-scan (*CrystalClear*; Rigaku, 2009 ▶) *T*_min_ = 0.962, *T*_max_ = 0.991
9386 measured reflections
1933 independent reflections
1668 reflections with *I* > 2σ(*I*)
*R*_int_ = 0.040

#### Refinement

*R*[*F* ^2^ > 2σ(*F* ^2^)] = 0.032
*wR*(*F* ^2^) = 0.079
*S* = 1.03
1933 reflections
193 parameters
1 restraint
H-atom parameters constrained
Δρ_max_ = 0.21 e Å^−3^
Δρ_min_ = −0.14 e Å^−3^

### 

Data collection: *CrystalClear* (Rigaku, 2009 ▶); cell refinement: *CrystalClear*; data reduction: *CrystalClear*; program(s) used to solve structure: *SHELXS97* (Sheldrick, 2008) ▶; program(s) used to refine structure: *SHELXL97* (Sheldrick, 2008) ▶; molecular graphics: *CrystalStructure* (Rigaku, 2009 ▶); software used to prepare material for publication: *CrystalStructure*.

## Supplementary Material

Crystal structure: contains datablocks global, I. DOI: 10.1107/S1600536810046465/is2629sup1.cif

Structure factors: contains datablocks I. DOI: 10.1107/S1600536810046465/is2629Isup2.hkl

Additional supplementary materials:  crystallographic information; 3D view; checkCIF report

## Figures and Tables

**Table 1:** Hydrogen-bond geometry (Å, °)

*D*—H⋯*A*	*D*—H	H⋯*A*	*D*⋯*A*	*D*—H⋯*A*
O1—H1*A*⋯O2	0.84	1.68	2.4701 (16)	155
C11—H11⋯O3	0.95	2.08	2.945 (2)	150

**Fig. 1.**
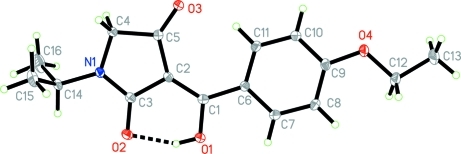
The molecular structure of the title compound, showing 30% probability displacement ellipsoids and the atom-numbering scheme.

## supplementary crystallographic information

### Comment

Many compounds containing the 3-acylpyrrolidine-2,4-dione moiety are novel heterocyclic compounds with antibiotic activity. Some of them are tenuazonic (Sticking, 1959), streptolydigin (Rinehart *et al.*, 1963), tirandamycin (Mackellar *et al.*, 1971), malonomycin (Baan *et al.*, 1978), alpha-cyclopiazonic acid (Sticking, 1959) and bata-cyclopiazonic acid (Holzapfel *et al.*, 1970). All these compounds possess a 3-acyltetramic acid moiety as a tricarbonylmethane structure and their hydrogen chemical shift of the enol hydroxy is about 11 p.p.m. (Wu *et al.*, 2002). On the other hand, most of the excellent inhibitors of *p*-hydroxyphenylpyruvate dioxygenase also possess similar characteristics, which are crucial for their bioactivity. Up to now, we have synthesized a series of 3-(un)substituted aroyl-1-benzylpyrrolidine-2,4-dione compounds and some of them have high herbicidal activity. The structure of the title compound, (I), helps us to investigate the relationship between structure and herbicidal activity.

The molecular structure of (I) is shown in Fig. 1. Atom H1A, involved in intramolecular hydrogen bonding between O1 and O3, was assigned to O1 rather than to O2, based on bond lengths. The C3—O2 distance is 1.267 (2) Å, which is longer than the C5—O3 distance of 1.222 (2) Å. In contrast, the C1—O1 distance [1.324 (2) Å] is intermediate between the normal carbonyl bond and the C—O single bond length (Allen *et al.* 1987). A similar situation has been found in 3-(1-hydroxyethylidene)-1- phenylpyrrolidine-2,4-dione, which contains the same pyrrolidine skeleton (Ellis & Spek, 2001).

### Experimental

The title compound was obtained according to the reported procedure of Matsuo *et al.* (1980). Colourless single crystals were obtained by recrystallization of the title compound from petroleum ether and ethyl acetate.

### Refinement

H atoms were placed in calculated positions, with C—H = 0.95–1.00 Å and O—H = 0.84 Å, and included in the final cycles of refinement using a riding model, with *U*_iso_(H) = 1.2*U*_eq_(C) and 1.5*U*_eq_(O). Friedel pairs were merged before the final refinement.

### Crystal data

**Table:**

C_16_H_19_NO_4_	*F*(000) = 308
*M**_r_* = 289.32	*D*_x_ = 1.307 Mg m^−^^3^
Monoclinic, *P*2_1_	Mo *K*α radiation, λ = 0.71075 Å
Hall symbol: P 2yb	Cell parameters from 2865 reflections
*a* = 11.3390 (15) Å	θ = 1.8–27.9°
*b* = 5.3830 (8) Å	µ = 0.09 mm^−^^1^
*c* = 12.0490 (18) Å	*T* = 113 K
β = 91.581 (7)°	Prism, colorless
*V* = 735.16 (18) Å^3^	0.42 × 0.26 × 0.10 mm
*Z* = 2	

### Data collection

**Table:**

Rigaku Saturn724 CCD diffractometer	1933 independent reflections
Radiation source: rotating anode	1668 reflections with *I* > 2σ(*I*)
multilayer	*R*_int_ = 0.040
Detector resolution: 14.222 pixels mm^-1^	θ_max_ = 27.9°, θ_min_ = 1.8°
ω scans	*h* = −14→14
Absorption correction: multi-scan (*CrystalClear*; Rigaku, 2009)	*k* = −7→7
*T*_min_ = 0.962, *T*_max_ = 0.991	*l* = −15→15
9386 measured reflections	

### Refinement

**Table:**

Refinement on *F*^2^	Primary atom site location: structure-invariant direct methods
Least-squares matrix: full	Secondary atom site location: difference Fourier map
*R*[*F*^2^ > 2σ(*F*^2^)] = 0.032	Hydrogen site location: inferred from neighbouring sites
*wR*(*F*^2^) = 0.079	H-atom parameters constrained
*S* = 1.03	*w* = 1/[σ^2^(*F*_o_^2^) + (0.0468*P*)^2^] where *P* = (*F*_o_^2^ + 2*F*_c_^2^)/3
1933 reflections	(Δ/σ)_max_ < 0.001
193 parameters	Δρ_max_ = 0.21 e Å^−^^3^
1 restraint	Δρ_min_ = −0.14 e Å^−^^3^

### Special details

**Table:**

Geometry. All e.s.d.'s (except the e.s.d. in the dihedral angle between two l.s. planes) are estimated using the full covariance matrix. The cell e.s.d.'s are taken into account individually in the estimation of e.s.d.'s in distances, angles and torsion angles; correlations between e.s.d.'s in cell parameters are only used when they are defined by crystal symmetry. An approximate (isotropic) treatment of cell e.s.d.'s is used for estimating e.s.d.'s involving l.s. planes.
Refinement. Refinement of *F*^2^ against ALL reflections. The weighted *R*-factor *wR* and goodness of fit *S* are based on *F*^2^, conventional *R*-factors *R* are based on *F*, with *F* set to zero for negative *F*^2^. The threshold expression of *F*^2^ > σ(*F*^2^) is used only for calculating *R*-factors(gt) *etc*. and is not relevant to the choice of reflections for refinement. *R*-factors based on *F*^2^ are statistically about twice as large as those based on *F*, and *R*- factors based on ALL data will be even larger.

### Fractional atomic coordinates and isotropic or equivalent isotropic displacement parameters (Å^2^)

**Table:**

	*x*	*y*	*z*	*U*_iso_*/*U*_eq_	
O1	0.70417 (9)	0.6075 (2)	0.35972 (9)	0.0257 (3)	
H1A	0.7435	0.6020	0.3017	0.039*	
O2	0.84775 (9)	0.4899 (2)	0.21845 (9)	0.0251 (3)	
O3	0.89526 (9)	−0.0316 (2)	0.53336 (9)	0.0251 (3)	
O4	0.48151 (10)	0.4135 (3)	0.82015 (9)	0.0299 (3)	
N1	0.96813 (11)	0.1603 (3)	0.26539 (10)	0.0203 (3)	
C1	0.74116 (13)	0.4313 (3)	0.42897 (13)	0.0193 (3)	
C2	0.83157 (13)	0.2766 (3)	0.39424 (13)	0.0186 (3)	
C3	0.88176 (13)	0.3211 (3)	0.28521 (13)	0.0192 (3)	
C4	0.99039 (13)	−0.0049 (3)	0.35867 (12)	0.0204 (3)	
H4A	1.0717	0.0165	0.3892	0.024*	
H4B	0.9791	−0.1805	0.3364	0.024*	
C5	0.89965 (13)	0.0726 (3)	0.44344 (13)	0.0192 (4)	
C6	0.67596 (12)	0.4261 (3)	0.53246 (13)	0.0188 (3)	
C7	0.59784 (13)	0.6206 (3)	0.55394 (13)	0.0220 (4)	
H7	0.5904	0.7537	0.5024	0.026*	
C8	0.53130 (13)	0.6236 (3)	0.64833 (13)	0.0233 (4)	
H8	0.4786	0.7570	0.6612	0.028*	
C9	0.54203 (13)	0.4304 (4)	0.72421 (13)	0.0227 (4)	
C10	0.61937 (14)	0.2358 (3)	0.70400 (14)	0.0248 (4)	
H10	0.6266	0.1029	0.7556	0.030*	
C11	0.68552 (14)	0.2340 (3)	0.60987 (14)	0.0236 (4)	
H11	0.7383	0.1004	0.5975	0.028*	
C12	0.39293 (16)	0.5968 (4)	0.83947 (16)	0.0361 (5)	
H12A	0.3344	0.6001	0.7769	0.043*	
H12B	0.4292	0.7635	0.8466	0.043*	
C13	0.3340 (2)	0.5264 (5)	0.94565 (16)	0.0554 (7)	
H13A	0.2992	0.3605	0.9377	0.067*	
H13B	0.2719	0.6472	0.9612	0.067*	
H13C	0.3926	0.5258	1.0070	0.067*	
C14	1.04354 (13)	0.1741 (3)	0.16876 (13)	0.0222 (4)	
H14	1.0084	0.2990	0.1160	0.027*	
C15	1.16687 (15)	0.2627 (5)	0.20292 (15)	0.0356 (5)	
H15A	1.1614	0.4220	0.2418	0.043*	
H15B	1.2141	0.2834	0.1366	0.043*	
H15C	1.2045	0.1397	0.2523	0.043*	
C16	1.0475 (2)	−0.0729 (5)	0.10959 (16)	0.0481 (6)	
H16A	1.0895	−0.1942	0.1567	0.058*	
H16B	1.0888	−0.0536	0.0397	0.058*	
H16C	0.9669	−0.1313	0.0939	0.058*	

### Atomic displacement parameters (Å^2^)

**Table:**

	*U*^11^	*U*^22^	*U*^33^	*U*^12^	*U*^13^	*U*^23^
O1	0.0261 (6)	0.0265 (7)	0.0247 (6)	0.0059 (5)	0.0053 (5)	0.0076 (6)
O2	0.0245 (6)	0.0277 (7)	0.0231 (6)	0.0011 (5)	0.0015 (4)	0.0074 (6)
O3	0.0269 (6)	0.0281 (7)	0.0206 (6)	0.0076 (5)	0.0045 (4)	0.0060 (6)
O4	0.0266 (6)	0.0393 (8)	0.0242 (6)	0.0102 (6)	0.0078 (5)	0.0036 (6)
N1	0.0208 (6)	0.0248 (8)	0.0155 (6)	0.0013 (6)	0.0028 (5)	0.0015 (6)
C1	0.0176 (7)	0.0179 (9)	0.0223 (8)	−0.0034 (6)	−0.0023 (6)	0.0004 (7)
C2	0.0179 (7)	0.0206 (9)	0.0174 (8)	−0.0034 (6)	0.0008 (6)	0.0015 (7)
C3	0.0180 (7)	0.0205 (9)	0.0191 (8)	−0.0042 (7)	−0.0010 (6)	0.0007 (7)
C4	0.0228 (7)	0.0208 (9)	0.0176 (7)	0.0019 (7)	0.0028 (6)	0.0019 (7)
C5	0.0194 (7)	0.0186 (9)	0.0196 (8)	−0.0019 (6)	0.0002 (6)	−0.0022 (6)
C6	0.0154 (7)	0.0198 (8)	0.0213 (7)	−0.0010 (7)	0.0008 (6)	−0.0005 (7)
C7	0.0218 (7)	0.0205 (9)	0.0235 (8)	0.0005 (7)	−0.0006 (6)	0.0019 (8)
C8	0.0209 (8)	0.0234 (9)	0.0256 (9)	0.0047 (7)	0.0008 (6)	−0.0038 (8)
C9	0.0170 (7)	0.0294 (9)	0.0219 (8)	−0.0005 (7)	0.0028 (6)	−0.0007 (8)
C10	0.0247 (8)	0.0241 (10)	0.0258 (9)	0.0038 (7)	0.0043 (7)	0.0048 (8)
C11	0.0200 (8)	0.0233 (9)	0.0277 (9)	0.0032 (7)	0.0044 (7)	0.0018 (8)
C12	0.0312 (9)	0.0451 (12)	0.0326 (10)	0.0143 (9)	0.0100 (8)	−0.0009 (10)
C13	0.0514 (13)	0.0781 (19)	0.0381 (12)	0.0272 (13)	0.0241 (10)	0.0097 (13)
C14	0.0225 (8)	0.0282 (10)	0.0162 (8)	−0.0014 (7)	0.0039 (6)	0.0007 (7)
C15	0.0266 (9)	0.0562 (14)	0.0244 (9)	−0.0101 (9)	0.0047 (7)	−0.0001 (10)
C16	0.0606 (14)	0.0485 (14)	0.0366 (11)	−0.0181 (12)	0.0245 (10)	−0.0199 (11)

### Geometric parameters (Å, °)

**Table:**

O1—C1	1.324 (2)	C8—H8	0.9500
O1—H1A	0.8400	C9—C10	1.392 (2)
O2—C3	1.2665 (19)	C10—C11	1.377 (2)
O3—C5	1.2222 (19)	C10—H10	0.9500
O4—C9	1.3635 (18)	C11—H11	0.9500
O4—C12	1.432 (2)	C12—C13	1.508 (3)
N1—C3	1.334 (2)	C12—H12A	0.9900
N1—C4	1.450 (2)	C12—H12B	0.9900
N1—C14	1.4654 (18)	C13—H13A	0.9800
C1—C2	1.394 (2)	C13—H13B	0.9800
C1—C6	1.467 (2)	C13—H13C	0.9800
C2—C5	1.459 (2)	C14—C16	1.510 (3)
C2—C3	1.466 (2)	C14—C15	1.523 (2)
C4—C5	1.528 (2)	C14—H14	1.0000
C4—H4A	0.9900	C15—H15A	0.9800
C4—H4B	0.9900	C15—H15B	0.9800
C6—C11	1.395 (2)	C15—H15C	0.9800
C6—C7	1.400 (2)	C16—H16A	0.9800
C7—C8	1.382 (2)	C16—H16B	0.9800
C7—H7	0.9500	C16—H16C	0.9800
C8—C9	1.388 (2)		
			
C1—O1—H1A	109.5	C11—C10—H10	119.7
C9—O4—C12	117.63 (14)	C9—C10—H10	119.7
C3—N1—C4	111.86 (12)	C10—C11—C6	120.77 (16)
C3—N1—C14	123.82 (14)	C10—C11—H11	119.6
C4—N1—C14	123.67 (12)	C6—C11—H11	119.6
O1—C1—C2	117.49 (14)	O4—C12—C13	107.10 (18)
O1—C1—C6	113.02 (13)	O4—C12—H12A	110.3
C2—C1—C6	129.47 (14)	C13—C12—H12A	110.3
C1—C2—C5	135.48 (14)	O4—C12—H12B	110.3
C1—C2—C3	118.59 (14)	C13—C12—H12B	110.3
C5—C2—C3	105.84 (13)	H12A—C12—H12B	108.5
O2—C3—N1	124.35 (14)	C12—C13—H13A	109.5
O2—C3—C2	124.57 (14)	C12—C13—H13B	109.5
N1—C3—C2	111.08 (13)	H13A—C13—H13B	109.5
N1—C4—C5	104.16 (13)	C12—C13—H13C	109.5
N1—C4—H4A	110.9	H13A—C13—H13C	109.5
C5—C4—H4A	110.9	H13B—C13—H13C	109.5
N1—C4—H4B	110.9	N1—C14—C16	110.85 (14)
C5—C4—H4B	110.9	N1—C14—C15	110.65 (12)
H4A—C4—H4B	108.9	C16—C14—C15	111.34 (16)
O3—C5—C2	132.20 (15)	N1—C14—H14	108.0
O3—C5—C4	120.79 (15)	C16—C14—H14	108.0
C2—C5—C4	107.01 (13)	C15—C14—H14	108.0
C11—C6—C7	117.94 (15)	C14—C15—H15A	109.5
C11—C6—C1	123.43 (14)	C14—C15—H15B	109.5
C7—C6—C1	118.61 (14)	H15A—C15—H15B	109.5
C8—C7—C6	121.55 (16)	C14—C15—H15C	109.5
C8—C7—H7	119.2	H15A—C15—H15C	109.5
C6—C7—H7	119.2	H15B—C15—H15C	109.5
C7—C8—C9	119.57 (16)	C14—C16—H16A	109.5
C7—C8—H8	120.2	C14—C16—H16B	109.5
C9—C8—H8	120.2	H16A—C16—H16B	109.5
O4—C9—C8	124.84 (15)	C14—C16—H16C	109.5
O4—C9—C10	115.63 (15)	H16A—C16—H16C	109.5
C8—C9—C10	119.53 (15)	H16B—C16—H16C	109.5
C11—C10—C9	120.63 (16)		
			
O1—C1—C2—C5	177.26 (17)	C2—C1—C6—C11	−9.0 (2)
C6—C1—C2—C5	−4.6 (3)	O1—C1—C6—C7	−9.19 (19)
O1—C1—C2—C3	1.3 (2)	C2—C1—C6—C7	172.57 (16)
C6—C1—C2—C3	179.52 (15)	C11—C6—C7—C8	−0.3 (2)
C4—N1—C3—O2	177.77 (14)	C1—C6—C7—C8	178.19 (14)
C14—N1—C3—O2	6.7 (2)	C6—C7—C8—C9	0.3 (2)
C4—N1—C3—C2	−2.23 (18)	C12—O4—C9—C8	5.8 (2)
C14—N1—C3—C2	−173.26 (13)	C12—O4—C9—C10	−174.26 (16)
C1—C2—C3—O2	−1.0 (2)	C7—C8—C9—O4	179.75 (15)
C5—C2—C3—O2	−178.00 (14)	C7—C8—C9—C10	−0.2 (2)
C1—C2—C3—N1	179.02 (14)	O4—C9—C10—C11	−179.68 (14)
C5—C2—C3—N1	2.00 (17)	C8—C9—C10—C11	0.3 (2)
C3—N1—C4—C5	1.48 (17)	C9—C10—C11—C6	−0.4 (2)
C14—N1—C4—C5	172.53 (14)	C7—C6—C11—C10	0.4 (2)
C1—C2—C5—O3	2.1 (3)	C1—C6—C11—C10	−178.04 (14)
C3—C2—C5—O3	178.34 (17)	C9—O4—C12—C13	176.02 (16)
C1—C2—C5—C4	−177.28 (17)	C3—N1—C14—C16	−129.11 (18)
C3—C2—C5—C4	−1.00 (16)	C4—N1—C14—C16	60.9 (2)
N1—C4—C5—O3	−179.62 (14)	C3—N1—C14—C15	106.86 (18)
N1—C4—C5—C2	−0.19 (16)	C4—N1—C14—C15	−63.1 (2)
O1—C1—C6—C11	169.26 (14)		

### Hydrogen-bond geometry (Å, °)

**Table:**

*D*—H···*A*	*D*—H	H···*A*	*D*···*A*	*D*—H···*A*
O1—H1A···O2	0.84	1.68	2.4701 (16)	155
C11—H11···O3	0.95	2.08	2.945 (2)	150
